# An Adaptive Source-Channel Coding with Feedback for Progressive Transmission of Medical Images

**DOI:** 10.1155/2009/519417

**Published:** 2009-01-26

**Authors:** Jen-Lung Lo, Saeid Sanei, Kianoush Nazarpour

**Affiliations:** ^1^Centre of Digital Signal Processing, School of Engineering, Cardiff University, Cardiff CF24 3AA, UK; ^2^Behavioural Brain Sciences, School of Psychology, University of Birmingham, Birmingham B15 2TT, UK

## Abstract

A novel adaptive source-channel coding with feedback for progressive transmission of medical images is proposed here. In the source coding part, the transmission starts from the region of interest (RoI). The parity length in the channel code varies with respect to both the proximity of the image subblock to the RoI and the channel noise, which is iteratively estimated in the receiver. The overall transmitted data can be controlled by the user (clinician). In the case of medical data transmission, it is vital to keep the distortion level under control as in most of the cases certain clinically important regions have to be transmitted without any visible error. The proposed system significantly reduces the transmission time and error. Moreover, the system is very user friendly since the selection of the RoI, its size, overall code rate, and a number of test features such as noise level can be set by the users in both ends. A MATLAB-based TCP/IP connection has been established to demonstrate the proposed interactive and adaptive progressive transmission system. The proposed system is simulated for both binary symmetric channel (BSC) and Rayleigh channel. The experimental results verify the effectiveness of the design.

## 1. Introduction

Transmission of medical images aiming at medical consultation, diagnosis, and treatment, or for training purposes demands highly reliable and high-speed communication systems. Since, typically, medical images contain large amounts of crucial clinical data, no visible encoding error is tolerated in the clinically important regions. Hence, advanced methodologies for gaining distortion-less reconstructed images, in fairly acceptable transmission rate, make it possible to transmit a medical image over a noisy channel. However, even by utilizing current high-speed broadband connections, the transmission of medical images has not been entirely successful; although using more parity bits enables a higher protection of the data against channel noise, the transmission time and the complexity of the channel coding considerably increase. By prioritizing the image regions based on their clinical information contents in one hand, and establishing a variable parity length with regard to the state of the communication channel on the other hand a higher data speed and immunity against noise can be achieved.

The concept of image coding utilizing discrete wavelet transform (DWT) and embedded zerotree wavelet (EZW) has been initially proposed in [1] and various methods have been developed based on this idea [2–8]. In [2], an effective scheme for image compression has been proposed where the spatial-spectral features of the image have been taken into account in order to show that wavelet transform is particularly well suited for progressive transmission. In [3], the concepts of DWT, EZW, progressive image transmission, and RoI have been utilized. These techniques have been effectively employed in our work. In [4], a method for progressive image transmission using EZW is presented where a number of constraints are imposed to provide variable bitrates for each frequency band. In [5], a compression method using linked significant tree (LST) has been utilized to encode all the significant coefficients in order to reduce the number of symbols and increase the compression efficiency. The authors in [6] have developed an image coding algorithm that incorporates the features of zerotree and zero-block based algorithms. The main contribution in this recent algorithm is partitioning the wavelet-transformed image into coefficient blocks and to generate roots in top-most subband by using a block tree. In [7], another progressive image transmission method has been proposed based on a quadtree segmentation procedure in order to provide fairly good quality transmitted images while keeping the computational cost low. The authors in [8] have developed strategies to exploit the wavelet coefficients in different subbands for designing different vector quantization (VQ) coding to achieve a fast and efficient progressive transmission.

Shannon's information theory states that the performance of transmission schemes can be optimized in source and channel coding separately. However, the result holds with infinite block size, infinite coding complexity, and stationary channels. Such conditions are difficult to meet in practice. Hence, joint source-channel coding (JSCC) scheme attracts the interest of many. The JSCC scheme consists of a quantizer, an entropy, and channel coders to meet the target source rate, to achieve the required robustness in channel coding, and to find an optimal bit allocation between source and channel coding systems. Several methods have focused on designing adaptive joint source-channel coding (JSCC) schemes and introducing the properties of unequal error protection (UEP) and rate allocation to achieve efficient transmission [9–20]. In [9], authors surveyed several algorithms in JSCC system design and fast source-channel bit allocation for various transmission channels. They also provided a local search strategy to improve the initial low-complexity and rate-optimal scheme to achieve a distortion-optimal solution. An image transmission system was proposed in connection with JPEG2000 [10, 11]. The JSCC and UEP schemes in this approach try to optimize the rate-distortion function to achieve an efficient transmission. JPEG2000 provides high compression efficiency and introduces various features not included in other compression algorithms. This method has also adopted a standard component for medical image compression by digital imaging and communications in medicine (DICOM). JPEG2000 adopts embedded block coding with optimal truncation (EBCOT) as a source coder. The EBCOT algorithm has high compression efficiency with a high complexity price, which needs a powerful central processing unit (CPU) for support. The compression part of the proposed scheme is similar to that of JPEG2000's. However, unlike JPEG2000's our scheme provides variable parity length for an adaptive channel coding, which replaces EBCOT with EZW for compression and progressive image transmission. In [12], a framework for solving the end-to-end problem of progressive transmission of images over noisy channels has been presented which allows finding the optimal length of parity codes for each fixed length package to have minimum distortion in the decoded images. In [13], a joint source-channel decoder-based method for data transmission over noisy channels has been introduced. Multipath fading for communication over a noisy channel has become a complex problem. The authors in [13] have used the maximum a posteriori (MAP) method to design a joint source-channel coding system. An adaptive source-channel coding scheme based on subband coding has been used in [14]. In this approach, a suitable source and channel coding rates for each subblock has been proposed to minimize the total distortion. In [15], a low-complexity technique for the transmission of medical images has been proposed whereby the channel noise information has been effectively exploited. The main idea in [15] is to segment the bitstream into consecutive subblocks of variable lengths and consider a tradeoff between the levels of source and channel coding systems. The authors in [16] proposed a JSCC method for transmission of images over fading channels and demonstrated the application of rate-compatible low-density parity-check (RC-LDPC) codes constructed by the progressive edge-growth algorithm, and used the UEP to protect the images. In [17], a parametric methodology in progressive source-channel coding for rate allocation has been developed. The channel code rate is chosen based on the properties of source coder and the conditions of channel. The UEP strategies for efficient progressive transmission are proposed in [18]. Under the condition of a target transmission rate, the JSCC algorithm computes a UEP scheme that maximizes the number of corrected bits over a noisy channel. In [19], authors have used a concatenation of rate-compatible punctured convolution code and cyclic redundancy check (CRC) code to form a UEP scheme and find the optimal rate allocation solutions for progressive image transmission.

Many communication systems allow two-way communication implying that the signals are back from receiver to transmitter to adjust the system parameters and obtain better system performance. The authors in [20–23] have utilized the concept called hybrid automatic repeat request (HARQ) to ask for retransmission of erroneously received data and tradeoff allocation between the source and channel codes according to a rate-distortion optimization policy. Many researches on tradeoff allocation bits between source codes and channel codes assume that the noise-level in the channel is known in advance. Therefore, the feedback signal is figured out based on the known noise levels and the constraints set by the user. In the proposed algorithm, this has been modified since the parity lengths change according to the noise level in the received data whereby the amount of detected incorrect data is used to predict the conditions of the practical transmission channel. For medical image transmission, the quality of the reconstructed images (especially in the RoI) should be acceptable. This can be set as the constraint for the quantizer and the compression algorithm in advance. Therefore, the quality of the reconstructed image is only affected by the channel state and the proximity to the image RoI. The feedback signal in the proposed scheme updates the parity length without the need for retransmission of the data or adding any extra overhead.

The principal idea behind all these methods is that in a progressive transmission framework, the receiver reconstructs the transmitted image at various bit rates, which makes the fast and reliable retrieval of large images possible. In other words, the quality of the reconstructed image totally depends upon the volume of the received data, and the images can be reconstructed in any bitrate. Furthermore, the image subblocks are coded separately. Due to the high sensitivity to transmission noise, progressive transmission of images over noisy channels has to be accompanied by an appropriate channel coding, or a joint source-channel coding scheme [12]. The noise in the current communication systems can be due to the electronic components, fading, Doppler shift for mobile systems, bad weather, interferences, attenuations, and so forth.

The Reed-Solomon (RS) codes utilized here are block-based error correcting codes and are widely used for channel coding. The RS(*p*, *q*) codes correct the symbol error and not the bit error; lengths in terms of symbols. Thus, RS is suitable for burst error detection and correction [23].

## 2. Joint Source-Channel Coding

In this paper, we propose a novel interactive and adaptive joint source-channel coding with feedback algorithm for progressive transmission of medical images. This approach benefits from the idea of the JSCC, RoI, UEP, and feedback technique together as follows.

1. The conventional RS channel coding has been used.
2. The variability of the parity code corresponds to both the proximity to the center of RoI and the state of the practical transmission channel at the same time; this makes an efficient source-channel coding possible.
3. The selectivity of the RoI is totally interactive and can be defined by the user in the receiver. This makes the method favorable to be used by clinicians who require fast access to the RoI in the image.
4. An algorithm for detection of the blocks in error is developed to detect and recover the corrupted data, estimate the noise level in the practical transmission channel, and feedback the information of the noisy channel to the transmitter to control the error rate in the reconstructed images in the subsequent transmission.

**Figure 1:**
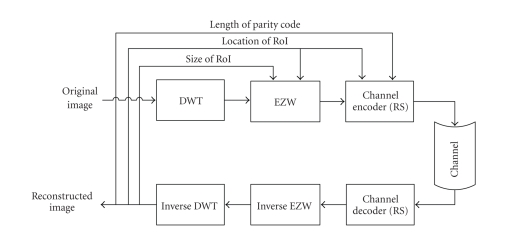
The overall system block diagram.

By utilizing our flexible system, a minimum distortion of the transmitted images in a fairly shorter transmission time is achieved. As the main contribution of this research, we adaptively control the lengths of parity code streams simultaneously with respect to the selected region (i.e., longer lengths correspond to the areas closer to the center of RoI) and the amount of corrupted received data in the receiver. The system block diagram is shown in Figure 1.

This paper is organized as follows. Section 3 briefly describes the concepts of DWT and EZW. Following that, we provide the details of RoI selection. In Section 2, application of RS channel coding in a variable-parity length scheme will be explained. In Section 4, an algorithm for detection of the blocks in error is developed to evaluate the amount of incorrect received data in the receiver. Simulation results are subsequently reported in Section 5 followed by concluding remarks in Section 6.

## 3. Discrete Wavelet Transform and Embedded Zerotree Wavelet

Wireless transmission of medical images involves construction of an effective joint source-channel coding to not only preserve the diagnostic information but also to enable progressive streaming of the data from the host to the receiver. EZW is a simple, efficient, and flexible compression algorithm for low bitrate image coding. The properties of DWT and EZW allow us to code and compress the data blocks individually and also compress it at any bitrate. Therefore, based on progressive encoding, we can compress a block into a bitstream with increasing accuracy. Traditionally, the input images are decomposed into many subblocks each to be coded, compressed, and transmitted individually. Therefore, the input image is segmented into a number of subblocks firstly. And then wavelet transform (WT) decomposes each subblock into different time-frequency components.

As it is detailed in [24], EZW codes the image into streams of six symbols, namely, *p*, *n*, *z*, *t*, 0, and 1. In mathematical terms, considering the image amplitude at location (*x*, *y*) is denoted by *γ*(*x*, *y*), and *t*_*n*_ is the threshold in *n*th iteration, the definitions of the symbols are given in Table 1. EZW suits progressive data transmission since it enables hierarchical encoding and decoding.

**Figure 2:**
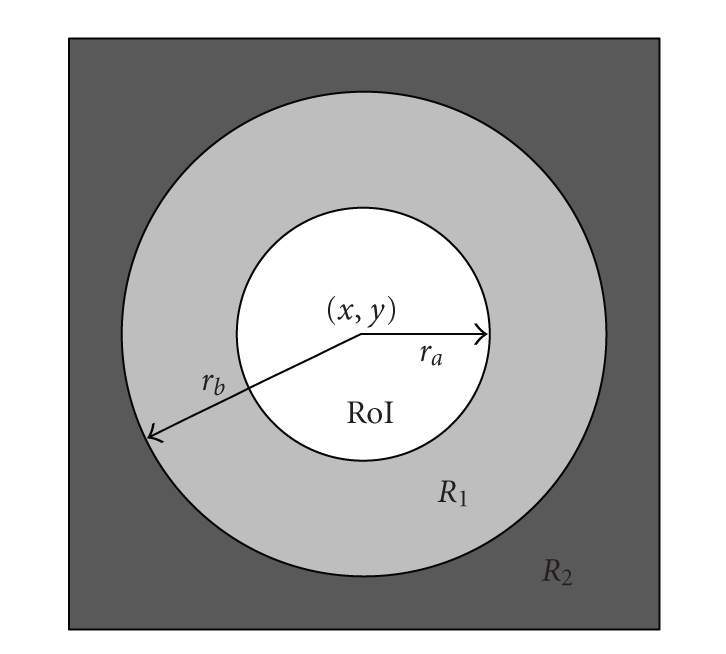
Areas of different priorities in an image centered at the center of RoI.

In the proposed system, we chose a 3-level Haar wavelet transform (HWT) to perform the DWT for each subblock due to its simplicity and being faster and easier to implement in comparison with other DWT methods [21]. The coefficients in the lowest frequency subbands show the background information of the subblocks. The coefficients in the higher frequency subbands represent the details and edges. After computing the HWT, we compress the coded data according to a variable thresholding mechanism governed by the EZW. Hence, a suitable approach is to use a variable threshold and transmit only those coefficients to the decoder that are larger than the threshold. The first step in the EZW algorithm is to determine the initial threshold level *t*_0_ and then repeatedly lowering the threshold by half at a time until the threshold has become smaller than the smallest coefficient to be transmitted; or the iteration is stopped by request. The initial threshold *t*_0_ is set as follows:

(1) $$t_{0}=2^{N},\;N=\log_{2}\max\left(\left|\gamma \left(x,y\right)\right|\right),$$

where max(·) refers to the maximum value. The final threshold level determines the length of the bitstream output through the EZW process, the compression ratio of the input images, and the resolution of the reconstructed image. The length of the output bitstream *M*_*i*_ is related to the number of times the threshold is halved as

(2) $$M_{i}=\overset{n_{Ti}}{\underset{k=0}{\sum }}B\left(\frac{t_{0i}}{2^{k}}\right),$$

where *B*(*t*) is the output bitstream of EZW based on the threshold *t*. *t*_0*i*_ is the initial threshold in the *i*th subblock, and *n*_*Ti*_ is the number of times the threshold is halved in the *i*th subblock. Therefore, potentially, we can achieve any resolution in the reconstructed images through setting the initial threshold, and the number of times the threshold is halved. As an example, Figure 2 shows the three regions of RoI, *R*_1_, *R*_2_ centered at point (*x*, *y*). In Figure 2, the resolution in area *R*_2_ is the lowest and that of RoI is the highest. Therefore, the quality of the reconstructed subblocks and consequently the compression rate depends on the size of the embedded zerotree. This is set based on the distance from the center of RoI. Based on the assigned parameters for EZW, the data in each subblock would be compressed with different rates depending on the location of the subblock. Often, the physician is only interested in a particular part of the image. Therefore, the system is designed in such a way to enable changing the location and size of the RoI without any emphasis on the other regions. In this example, the RoI, *R*_1_, and *R*_2_ may be defined as

for RoI:
  (3) $$\sqrt{{\left(i-x\right)}^{2}+{\left(j-y\right)}^{2}}\leq r_{a},$$
for *R*_1_:
  (4) $$r_{a}<\sqrt{{\left(i-x\right)}^{2}+{\left(j-y\right)}^{2}}\leq r_{b},$$
for *R*_2_:
  (5) $$\sqrt{{\left(i-x\right)}^{2}+{\left(j-y\right)}^{2}}>r_{b},$$

where *x* and *y* are the coordinates of the center of RoI assigned by the physician using a mouse click, thereafter both values are sent back to the host. The values of *r*_*a*_ and *r*_*b*_ are the radius of RoI and *R*_1_, and *i* and *j* express the *x* − *y* image pixels' coordinates. In the successive progressive stages, the values of *r*_*a*_ and *r*_*b*_ gradually expand in the next progressive transmission to create the reconstructed image of higher quality. In our proposed algorithm, the first transmitted image is the background low-resolution image. Then, the reconstruction is progressively continued regarding the highest priority of RoI.

The progressive bitstream is heavily affected by the channel noise; a single bit error may make the reconstruction of the image impossible [10]. The adaptive joint source-channel coding and the developed blind technique for evaluation of errors in the receiver are the effective ways to address this problem. The channel coding variables can be adjusted by using the feedback information about the channel state and the image region content. The RS code is a subset of BCH codes; they are linear block codes and are efficient for bursty-type transmission channels. The RS codes are constructed by considering a polynomial for the input information and then use the polynomial coefficients instead of the original data for transmission. In an RS(*p*, *q*) code, *p* = 2^*m*^ − 1 is the number of symbols in a codeword, and *m* is the number of bits in each symbol. In the proposed algorithm, the data in different image regions, as denoted in Figure 2 for three regions, are protected by variable length parity codes as for the UEP. The data in the RoI is treated as the most important information and protected by longer length parity codes. The rest of the data is protected by shorter parity codes.

**Figure 3:**
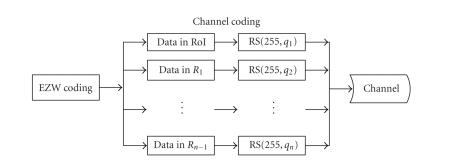
The transmitter including the proposed channel coding block diagram.

**Figure 4:**
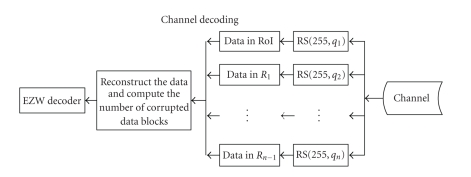
The block diagram for the receiver.

Figure 3 illustrates the channel coding strategy and Figure 4 shows the receiver. The overall channel code length remains fixed and the length of message *k*, *q*_*k*_; *k* = 1, 2,…, *n*, and the parity length *C* are variable. For RS codes, (255 − *q* *n*)/2 indicates the error-correction capability of RS coder. Here, the RS codes, RS(255, *q*), have 255 symbols in length. According to the UEP, the ratio of parity to overall code length for the *n* regions should follow

(6) $$C_{\text{RoI}}>C_{R_{1}}>C_{R_{2}}\cdots >C_{R_{n-1}},$$

where *C*_RoI_, the length of parity code, is for the RoI and so on. Furthermore, the length of parity code is also affected by the noise in the channel, that is, *C*_region_~(*r*, *N*), where *r* is the distance from the center of RoI, and *N* expresses the noise in the practical transmission channel.

An adaptive variable parity allocation requires the error between the transmitted image *I*(*x*, *y*) and the received image $\hat{I}(x,y)$ to be minimized under the desired conditions. Suppose that the error is defined as

**Figure 5:**
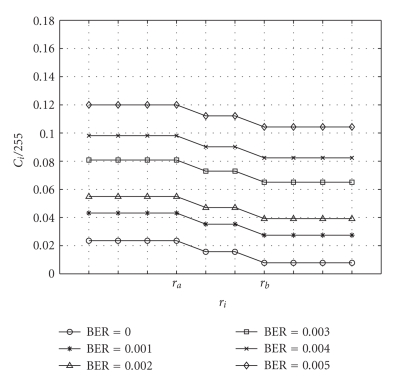
The ratios between the lengths of parity code *C*_*i*_ and overall codeword of 255 symbols in different regions at various fixed noise levels.

**Figure 6:**
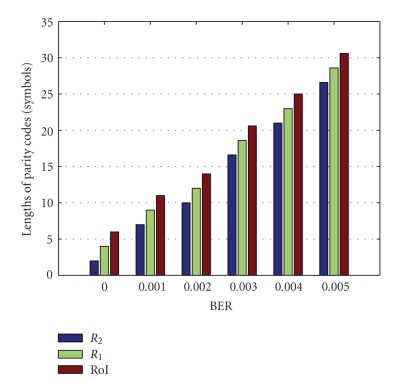
Lengths of the parity codes based on various channel noise levels.

(7) ε=∥I(x,y)−I^(x,y)∥2,

where ||·||_2_ denotes the *I*_2_-norm. Generally, we wish to have the optimum parity length such that

(8) $$C_{\text{opt}}=\underset{C}{\min}\;\varepsilon \;\;\text{subject}\;\;\text{to}\;\;\varepsilon \leq \varepsilon_{T},$$

where *ε*_*T*_ is an acceptable error level in the receiver. According to the above discussion, the parity length can be defined as

(9) $$C=g\left(r,\frac{S}{N}\right)=f(r,\text{BER),}$$

where *S*/*N* and BER stand, respectively, for signal-to-noise ratio and bit-error rate (the BER here represents the noise situation in the channel and does not refer to the output bit-error rate). The functions *g* and *f* are generally nonlinear functions, which can be defined empirically based on a number of trials. From Figure 5, *f*~(*α*_0_ − *α* *r*) for a fixed BER, where *r* is measured with respect to the number of pixels, and from Figure 6, it can be concluded that *f*~(*β* BER + *β*_0_) for fixed proximity distance *r*. In more general applications, a more accurate and possibly complicated function may be adopted. Therefore, a reasonably accurate function can be modeled as

(10) $$f(r,\text{BER)}=(\alpha_{0}-\alpha r)(\beta \;\text{BER}+\beta_{0}),$$

or

(11) $$f(r,\text{BER)}=\mu_{3}\text{BER}-\mu_{2}r\cdot \text{BER}-\mu_{1}r+\mu_{0},$$

where *μ*_*i*_s are constant coefficients and can be easily found based on Figures 5 and 6.

## 4. Detection of the Blocks in Error

Embedded coding has many advantages, especially for progressive image/video transmission, because the reconstructed images can be decoded at any bitrate. However, it is highly sensitive to transmission noise and frequently collapses if even a single transmitted information bit is incorrectly decoded. In most cases, the receiver sends back a signal called HARQ to the transmitter requesting for retransmission under the new constraints. Here, the channel noise level has to be somehow estimated in the receiver. This will enable the change in the parity length in the following transmissions. The estimation process is blind since there is no a priori information about the channel. In some practical cases however, a test image can be transmitted occasionally and evaluated in the receiver.

In the proposed scheme, we have developed an algorithm to detect the corrupted data in the receiver. This algorithm detects the address in which the number of symbols for each subblock is indicated. The algorithm reassigns the number of symbols to each subblock according to a built-in decision making criterion (policy) when the number of symbols within the received data is determined incorrectly by the receiver. Since the header information provides the number of symbols per each data package, an extra check can be carried out to ensure that the header is divisible by 4 and is not greater than 64 (to address each pixel) for the 8 × 8 subblocks. For more accuracy, the algorithm checks the next address of the number of symbols to ensure that the current data is correct. If successive error data is detected, the algorithm is able to determine the number of subblocks and reassign the number of symbols for each subblock in the set of detected incorrect data. Although, the proposed algorithm does not have capability of recovery of the current corrupted package, but it can conjecture an appropriate number to replace the corrupted data based on the built-in decision making criterion (policy) to avoid the reconstructed image to collapse. This adjusts the system for the transmission of the subsequent package and prevents propagation of the error. This not only enhances the quality of the reconstructed image but also provides a feedback for the adjustment of parity length.

The performance of the system in different noise-levels is evaluated using the peak signal-to-noise ratio (PSNR) defined as

(12) $$\text{PSNR}(\text{dB})=10\;{\log\;}_{10}\frac{A^{2}}{\text{MSE}},$$

where *A* is the maximum image amplitude, and MSE is defined as

(13) MSE=∥I^−I∥22∥I∥22,

where $\hat{I}$ represents the reconstructed subblock of the image, and *I* is the subblock of the original input image.

Finally, the parallel structure of the channel coding and decoding scheme is very suitable in hardware implementation of the system. A number of parallel boards can be easily used in order to speed up the overall process.

## 5. Simulation Results

In this work, the proposed system is simulated in both binary symmetric channel (BSC) and flat-fading Rayleigh channel. The BSC is the simplest channel model, only zeros and ones are conveyed in the channel. Therefore, we can simplify the analysis and enable a fast software implementation. But the wireless mobile communication channels are often considered to be with flat-fading Rayleigh noise. In this paper, we simulated both BSC and flat-fading channel models and tested the performance of the proposed techniques against both models. RS(255, *q*) is used in the proposed scheme. The RS codes correct the symbol error and not the bit error. The noise in the simulated channel has been considered in such a way to set a BER of 0.01 in the received end. For when the errors are uniformly distributed, the average parity length is 42 for a 255 length code length. This recovers the RoI perfectly when either BSC or Rayleigh channel is considered and the channel noise is equivalent to BER = 0.01.

The developed algorithm has been tested for a number of images, two of which are analyzed here. The first image is a 150 × 123 pixels color dental implant image, and the second image is a 508 × 512 pixels monochrome X-ray bone image. Both are to be transmitted via TCP/IP. The proposed system follows the diagram in Figure 1. Each noisy channel involves a binary symmetric channel (BSC) and flat-fading Rayleigh channel with a certain BER. In this simulation, the error bits are generated by randomly inverting a certain percentage of bits in the EZW bitstream. To verify the effectiveness of the system, the image regions are progressively transmitted through four stages of *P*_1_, *P*_2_, *P*_3_, and *P*_4_. During *P*_1_, the background image is transmitted. *P*_2_ and *P*_3_ are the second and third stages, both for transmission of RoI, *R*_1_, and *R*_2_. *P*_4_ is the fourth stage mainly to transmit the details of the RoI (and the rest of the image if necessary). In the approach presented here, firstly, the user (physician) specifies the address of the transmitted medical image in the transmitter within the dialogue-based software. After receiving the low-resolution background image, the user identifies the center of RoI by a mouse and the radius of RoI by entering a value within the dialogue-based software. Then, the algorithm adjusts the length of the parity codes initially proportional to the proximity of the image regions to the center of RoI as *C*_0_, *C*_0_ − 2, *C*_0_ − 4 for RoI, *R*_1_, and *R*_2_, respectively.

Accordingly, the receiver detects and counts the packages in error by estimating the status of the channel. The parity lengths remain the same if the distortion in the reconstructed image is acceptable. Otherwise, the codes will be adjusted automatically. Typically, the ratio of parity code to the overall code length is larger for the clinically higher priority areas, that is, the areas closer to the center of RoI as stated in (6). Figure 5 indicates the ratios of parity length and the overall codeword according to the experimental results.

Table 2 gives an example of the percentage of information for the regions *R*_1_, *R*_2_, and *R*_2_ for fixed proximity levels of *r*_*a*_ and *r*_*b*_, as in Figure 9. Table 3 indicates the average compression ratios for various regions before the channel coding during the four successive transmission stages. The compression ratio is defined as the ratio between the data volume of the coded data and the original data. However, by changing one of RoI coordinates, or *r*_*a*_ and *r*_*b*_, data in Tables 2 and 3 are also changed.

In Figure 6, the parity lengths are found by averaging the results of 10 trials under various noise levels. These are estimated by the algorithm developed for detection of the blocks in error in the receiver. Data in RoI is the most important data in the overall image; therefore, the length of parity codes is longer than that in *R*_1_ and *R*_2_. In the proposed system, the codeword length of RS codes is 255, and the number of error bit is generated at random.

**Figure 7:**
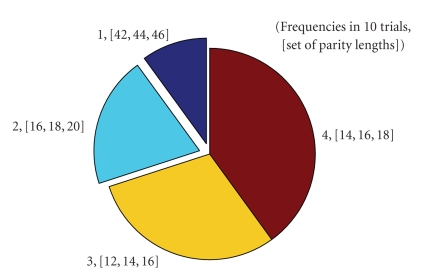
Average distribution (frequency) of the set of parity lengths in 10 trials for BER = 0.003.

Figure 7 shows the frequency of the set of parity lengths in 10 trials for when the channel noise is set by BER = 0.003 equivalent to the occurrence of 7 errors. As long as the error in the receiver remains higher than a threshold *t*_*h*_, the length of parity increases. Consequently, if the error falls below a level *t*_*l*_ < *t*_*h*_ the parity length increases. These threshold levels are empirically selected by following the constraint in (8). In these cases, the parameters in (10) and (11) are approximately *α*_0_ = 0.08, *α* = 2 × 10^−4^, *β*_0_ = 5, *β* = 5 × 10^3^ and accordingly *μ*_0_ = *α*_0_ *β*_0_ = 0.4, *μ*_1_ = *α* *β*_0_ = 10^−3^, *μ*_2_ = *α* *β* = 1, and *μ*_3_ = *α*_0_ *β* = 4 × 10^−2^.

**Figure 8:**
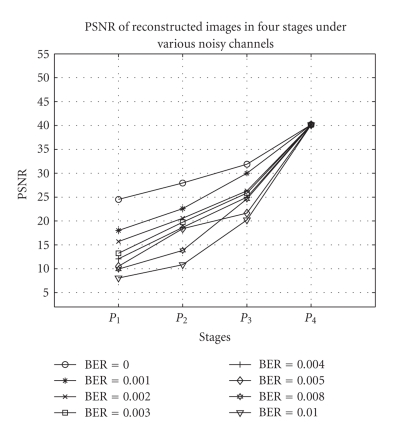
PSNR for successive transmission of four stages at different BERs under various noise-level conditions.

**Figure 9:**
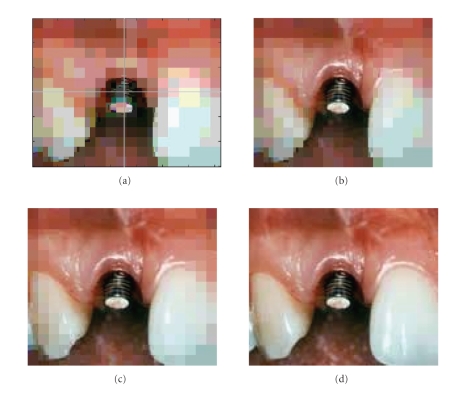
The transmitted image over the low noise: (a) the background image at stage *P*_1_ and the location of RoI in the center of the image, (b) the transmitted image after stage *P*_2_, (c) the transmitted image after stage *P*_3_, and (d) shows the completely decoded image after stage *P*_4_.

**Figure 10:**
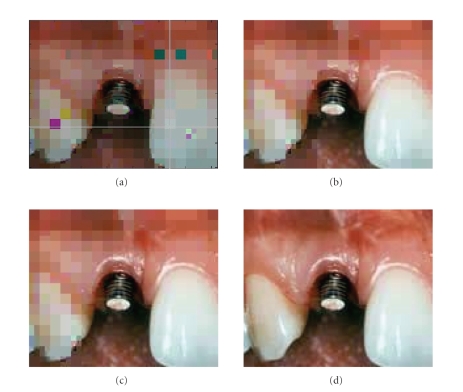
Similar results as in Figure 9 when the RoI is selected in the corner of the image.

**Figure 11:**
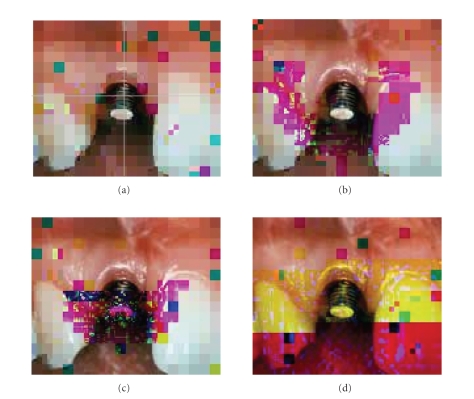
An example of quality of the decoded image with fixed length parity: (a) background image with the location of RoI in the center, (b) the image reconstructed at stage *P*_2_; several subblocks in error in the area of RoI and some error subblocks in the vicinity of RoI, (c) the number of subblocks in error increases when the volume of the data in the receiver increases, that is, the resolution of the higher-priority regions increases, and (d) the complete transmitted image.

**Figure 12:**
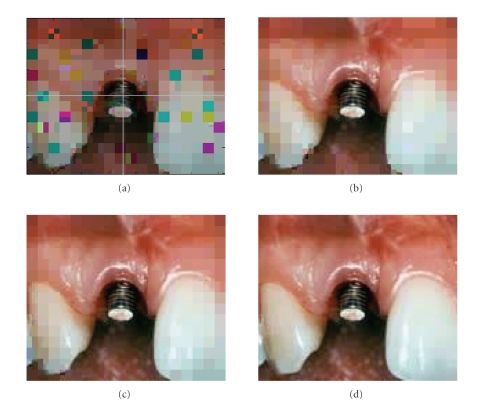
The decoded image with variable length of parity codes over the noisy channel. (a) A background image and the location of RoI selected in the center of image reconstructed after stage *P*_1_, (b) the reconstructed image after stage *P*_2_, no error subblocks are found in the reconstructed image because the lengths of parity codes are adjusted automatically based on the previous volume of incorrect data in the receiver, (c) the reconstructed image after stage *P*_3_; the lengths of parity bits in stage 3 are as same as in stage 2 because no incorrect data was found in the reconstructed image after stage 2, (d) the complete transmitted image with no error subblocks.

**Figure 13:**
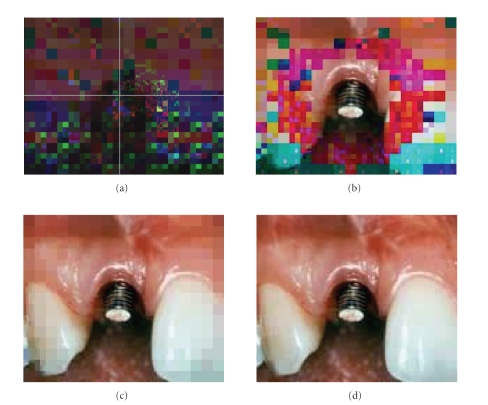
A decoded image with variable length of parity codes over noisy channel: (a) several error subblocks are detected in stage 1, (b) several error subblocks still are found in stage 2 indicating that the feedback message is incorrect or the channel condition becomes noisier. However, RoI is still error-free based on the UEP. (c) No error subblock is detected in stage 3 because the length of parity is adjusted again according to previous channel state, although there can still be some error. (d) The complete transmitted image with no error subblocks.

**Figure 14:**
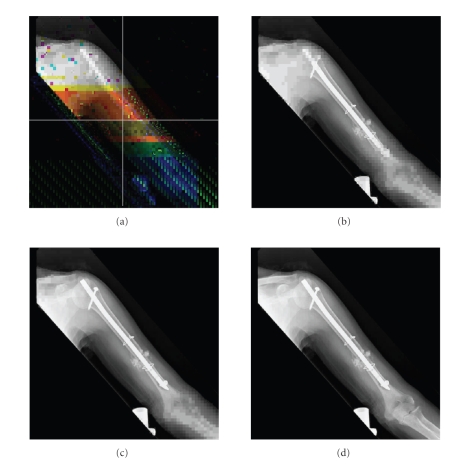
As another example, a decoded image with variable length parity, which is a 508 × 512 pixels monochrome X-ray bone image: (a) a background image and the location of RoI selected in the center of image reconstructed after stage *P*_1_; many subblocks are in error in the background image, (b) the reconstructed image after stage *P*_2_, no error subblock is found in the reconstructed image. (c) The reconstructed image after stage *P*_3_, and (d) the complete transmitted image having no error subblocks.

Figure 8 shows the PSNRs for successive transmission of four stages under various noise-level conditions. Figure 9(a) shows the background image sent during *P*_1_ stage. Figure 9(b) is progressively reconstructed image after stage *P*_2_ in which the RoI, *R*_1_, and *R*_2_ are reconstructed. At this stage, the center of RoI is denoted by the user via mouse click. Figure 9(c) represents the reconstructed image at stage *P*_3_ during which the regions RoI, *R*_1_, and *R*_2_ are reconstructed. The regions of RoI and *R*_1_ are gradually increased in resolution. Figure 9(d) is the final and complete image after stage *P*_4_. The same procedure can be followed for encoding and transmission of any other medical image. However, the coordinates of the center of RoI as well as the size of RoI maybe adjusted according to the requirement by the user. For example, in Figure 10, the RoI is selected in the corner. Figure 11 demonstrates that a fixed-size parity code is not suitable for an efficient transmission system. However, the system has been modified based on the proposed method in Sections 2 and 4 to allow variable lengths of parity. Figures 12 and 13 show no error in the RoI stating that the overall system has been remarkably improved. In Figure 14, another example of a decoded image (a 508 × 512 monochrome X-ray bone image) is given, and the variable length parity has been examined. The background image suffers from heavy noise. However, the transmission can continue until the last stage during which a complete error-free image is reconstructed.

## 6. Conclusion

In this paper, we presented a new adaptive source-channel coding with feedback for the progressive transmission of medical images. The system is adaptive to both image content and channel specifications. However, this application is merely for wireless channels (generally narrowband). The capability of data error detection and correction with automatic adjustment, low image transmission time, and efficient communication are the key features in this proposed system. Therefore, the length of parity codes can be adjusted automatically based on the location of the image subblocks and the practical characteristics of the communication channel to provide an adequate data protection. The overall code length for the channel encoder/decoder is fixed. This makes it easy for hardware implementation. A wide range of fluctuations in the channel characteristics (mainly noise level) can be tolerated in the system. The algorithm of detection of subblock in errors can detect the packages in error in the receiver and feed it back to the transmitter for adjustment of the parity length. The proposed system has also been tested for the communication channels with different capacities and noise levels. The presented results verify the effectiveness of the system in terms of both adaptivity and flexibility of interaction. A MATLAB-based TCP/IP connection has been established to demonstrate the proposed interactive and adaptive progressive transmission system. Although some theoretical results are comparable to those of other new techniques such as the UEP or rate allocation approaches, this contribution provides a practical, flexible, and interactive method which suits medical applications.

## Figures and Tables

**Table 1:** Definition of symbols.

Symbols	Description
*p*	For *γ*(*x*, *y*) ≥ *t*_*n*_, called *significant coefficients* at threshold *t*_*n*_.
*n*	For *γ*(*x*, *y*) < 0 & |*γ*(*x*, *y*)| ≥ *t*_*n*_, called *negative significant*.
*z*	For *γ*(*x*, *y*) < *t*_*n*_, but some of its descendants have a value greater than *t*_*n*_, called *isolated zero*.
*t*	For *γ*(*x*, *y*) < *t*_*n*_, and all its descendants have magnitudes less than *t*_*n*_, called *zerotree zero*.
1,0	Refinement bits for reconstructing image.

**Table 2:** The information percentage transmitted for each area during the stages *P*_1_ to *P*_4_ images.

Percentage of area	*P*_1_	*P*_2_	*P*_3_	*P*_4_
RoI	0%	18.15%	41.86%	100%
*R*_1_	0%	37.04%	44.07%	0%
*R*_2_	100%	44.81%	14.07%	0%

**Table 3:** The average compression ratio for various regions for the four stages of the progressive image transmission.

Compression ratios	*P*_1_	*P*_2_	*P*_3_	*P*_4_
Overall image	3.063	1.069	0.716	0.405
RoI	0	0.353	0.401	0.405
*R*_1_	0	1.232	1.814	0
*R*_2_	3.063	3.746	3.387	0

## References

[1] Shapiro JM (1993). Embedded image coding using zerotrees of wavelet coefficients. *IEEE Transactions on Signal Processing*.

[2] Antonini M, Barlaud M, Mathieu P, Daubechies I (1992). Image coding using wavelet transform. *IEEE Transactions of Image Processing*.

[3] Chapman C, Sanei S, Dilmaghani R, Said F Progressive transmission of medical images using embedded Zertree wavelet encoding.

[4] Dilmaghani RS, Ahmadian A, Ghavami M, Aghvami AH (2004). Progressive medical image transmission and compression. *IEEE Signal Processing Letters*.

[5] Muzaffar T, Choi T-S Embedded linked significant tree wavelet image coder.

[6] Moinuddin AA, Khan E Wavelet based embedded image coding using unified zero-block-zero-tree approach.

[7] Hu Y-C, Jiang J-H (2005). Low-complexity progressive image transmission scheme baesd on quadtree segmentation. *Real-Time Imaging*.

[8] Wu M-N, Lin C-C, Chang C-C A fast wavelet-based image progressive transmission method based on vector quantization.

[9] Hamzaoui R, Stanković V, Xiong Z (2005). Optimized error protection of scalable image bit streams [advances in joint source-channel coding for images]. *IEEE Signal Processing Magazine*.

[10] Wu Z, Bilgin A, Marcellin MW (2005). Joint source/channel coding for image transmission with JPEG2000 over memoryless channels. *IEEE Transactions on Image Processing*.

[11] Liu W, Daut DG (2007). Progressive image transmission based on joint source-channel decoding using adaptive sum-product algorithm. *EURASIP Journal on Image and Video Processing*.

[12] Yousefi'zadeh H, Jafarkhani H, Etemadi F Distortion-optimal transmission of progressive images over channels with random bit errors and packet erasures.

[13] Chebil J, Boashash B, Deriche M Combined source channel decoding for image transmission over fading channels.

[14] Srinivasan M, Chellappa R Joint source-channel subband coding of images.

[15] Sherwood PG, Zeger K Progressive image coding on noisy channels.

[16] Pan X, Banishashemi AH, Cuhadar A (2006). Progressive transmission of images over fading channels using rate-compatible LDPC codes. *IEEE Transactions on Image Processing*.

[17] Nosratinia A, Lu J, Aazhang B (2003). Source-channel rate allocation for progressive transmission of images. *IEEE Transactions on Communications*.

[18] Stanković VM, Hamzaoui R, Charfi Y, Xiong Z (2003). Real-time unequal error protection algorithms for progressive
image transmission. *IEEE Journal on Selected Areas in Communications*.

[19] Liu Z, Zhao M, Xiong Z (2005). Efficient rate allocation for progressive image transmission via unequal error protection over finite-state Markov channels. *IEEE Transactions on Signal Processing*.

[20] Chande V, Farvardin N, Jafarkhani H Image communication over noisy channels with feedback.

[21] Lu J, Nosratinia A, Aazhang B Progressive joint source-channel coding in feedback channels.

[22] Chande V, Farvardin N, Jafarkhani H Image communication over noisy channels with feedback.

[23] Zhai F, Eisenberg Y, Pappas TN, Berry R, Katsaggelos AK (2006). Rate-distortion optimized hybrid error control for real-time packetized video transmission. *IEEE Transactions on Image Processing*.

[24] Valens C EZW encoding. http://pagesperso-orange.fr/polyvalens/clemens/ezw/ezw.html.

